# Biological Restoration of a Fractured Anterior Tooth with the Use of Dentine Pin (Biopins)

**DOI:** 10.1155/2015/138474

**Published:** 2015-12-08

**Authors:** Lilian Capanema Nogueira, Karine Taís Aguiar Tavano, Nayara Kelly Lyrio Ferraz, José Cristiano Ramos Glória, Adriana Maria Botelho

**Affiliations:** ^1^The Federal University of Minas Gerais, Belo Horizonte, MG, Brazil; ^2^The Pontifical Catholic University of Minas Gerais, Belo Horizonte, MG, Brazil; ^3^The Federal University of the Jequitinhonha and Mucuri Valleys (UFVJM), Diamantina, MG, Brazil; ^4^The Bauru Dental College, Federal University of São Paulo, São Paulo, SP, Brazil

## Abstract

This case study describes the esthetic and functional reconstruction of a fractured maxillary central incisor. Due to the requirement for additional retention, treatment was performed using the homogenous technique of biological restoration associated with cementation of posts made from human dentin (biopins). This type of treatment is a new alternative to conventional techniques and favors the dental esthetic and function, as well as biocompatibility, and is an inexpensive procedure.

## 1. Introduction

Dental trauma occurs quite frequently and principally affects children and adolescents [1, 2]. The maxillary central incisors are the most susceptible teeth to fractures because of their vulnerable position in the dental arch [3, 4]. Traumatic injuries cause a significant impact on the quality of life of children in terms of physical and psychological discomfort and have the potential to negatively affect social relationships [5, 6].

Depending on the type and extent of the trauma, the reestablishment of the esthetic and function of a fractured tooth can be performed using a number of restorative methods: composite resin restorations and ceramic restorations, with or without additional retentions (intracanal or dental posts) [7, 8]. Whenever possible, the most commonly recommended technique is autogenous or homogenous fragment bonding [9, 10]. Fragment bonding is a procedure that can be labeled as “biological restoration,” consisting of the use of fragments of the fractured tooth (autogenous bonding) or fragments obtained from an extracted tooth (homogenous bonding) [11, 12]. This technique allows reestablishment of the dental element with its original characteristics and the maintenance of occlusion in natural teeth and is inexpensive and has a positive psychological effect, since the social and emotional well-being of the patient and their family is restored [13–15].

Restorative dental materials have reached high levels of development and stability although there is no material in existence that completely meets the requirements to restore the loss of tooth structure in terms of plastics, esthetics, and functionality [16, 17]. Whenever possible, fragment bonding is the most adequate restoration technique for fractured anterior teeth. The result is esthetically satisfactory in terms of translucence, opalescence, fluorescence, and the texture of the natural surface [18, 19].

When the remainder of the tooth is not sufficient to retain the fragment or the restorative material, it is necessary to use additional resources for retention in both vital and devitalized teeth [20]. It is possible to use prefabricated cemented, rubbed, or screwed interdental posts for devitalized teeth [21]. Cement posts are the most commonly used as they do not cause tension in the dentin [21]. Interdental posts of human dentin or “biopins” are one possible option for permanent anterior vital teeth that require additional retention [22]. Biopins provide a greater level of retention and stability in vital teeth and also have the following advantages: biocompatibility; resilience; coefficient of thermal expansion similar to dental element; being esthetically favorable; production of fewer cracks in the dentin; and being inexpensive [23].

An ex vivo study demonstrated a significant increase in fracture resistance among teeth restored with experimental dentin posts in comparison to teeth restored with fiber-reinforced composite posts. This suggests the possible reinforcing potential of the dentin post. However, there are no existing studies that test the effectiveness of dentine pins [24].

The aim of the present case study was to describe a new homogenous fragment bonding technique for vital teeth, associated with the cementation of interdental posts produced with human dentin (biopins).

## 2. Case Study

**Figure 1:**
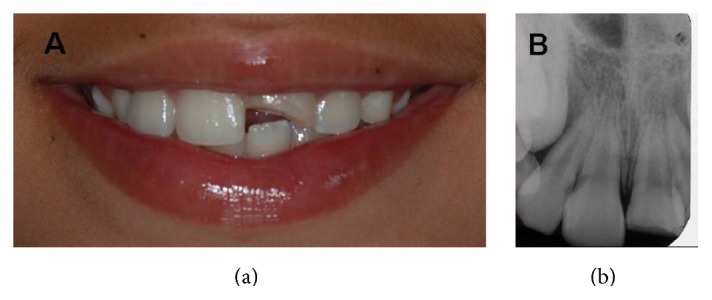
(a) Initial clinical aspect. (b) Initial radiographic aspect.

**Figure 2:**
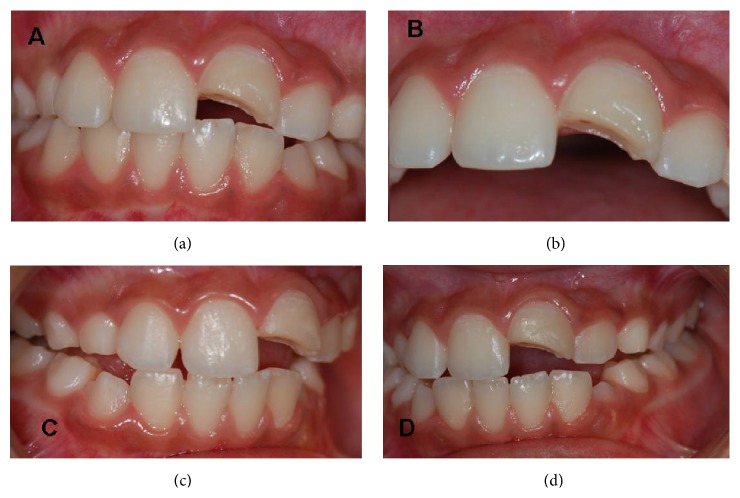
(a), (b) Initial clinical aspect, close-up. (c) and (d) Malocclusion in laterality.

A nine-year-old girl attended the dental clinic of the* Universidade Federal dos Vales do Jequitinhonha e Mucuri* (UFVJM), Diamantina, MG (Brazil), exhibiting a fracture in the middle third of the crown of tooth 21 (Figures 1(a), 2(a), and 2(b)). The guardian reported that the child had undergone previous unsuccessful treatments. The restorations had loosened during protrusive movements in the two years since the treatment. Clinical and radiographic examinations revealed fractured enamel and dentin without pulp involvement.

Since the child did not have the original fragment, a homogenous biological restoration procedure was proposed, associated with the use of dentine pins (biopins) in order to increase the retention of the fragment to the remainder of the tooth. The technique was explained in terms of esthetics, functional, and hygienic characteristics, and the guardian signed an informed statement of consent.

The technique was executed during two separate sessions. After anamnesis and clinical and radiographic examination, an assessment of the color and the incisal guides (Figures 2(c) and 2(d)), upper and lower arch molding was performed, as well as casting of the mold with a special type of plaster (*Durone Tipo* IV, Dentsply, Brazil) to enable the cutting of the tooth fragment and the production of the dentine pins in the laboratory.

**Figure 3:**
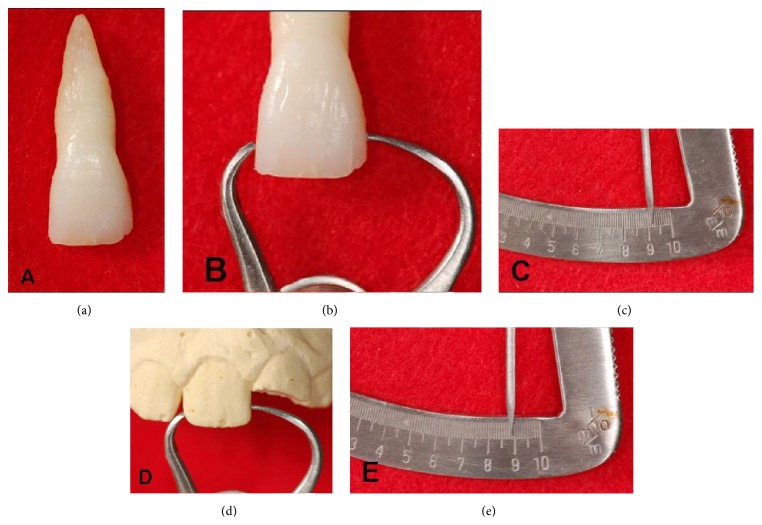
(a) Selected extracted central incisor. (b), (c) Mesiodistal measurement of the extracted tooth. (d), (e) Mesiodistal measurement of the remaining tooth in the laboratory.

**Figure 4:**
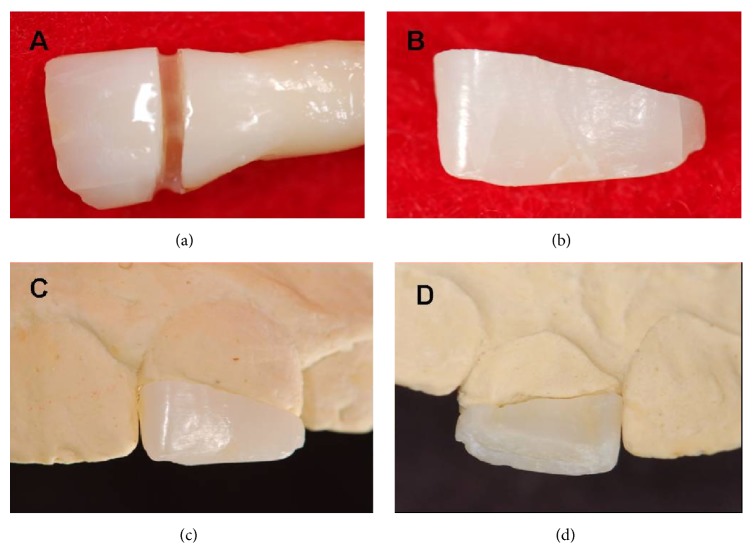
(a) Cut of the dental element to obtain the restorative fragment. (b) Cut of dental fragment. (c) Adaptation of the fragment to the plaster model, vestibular view. (d) Adaptation of the fragment to the plaster model, palatal view.

**Figure 5:**
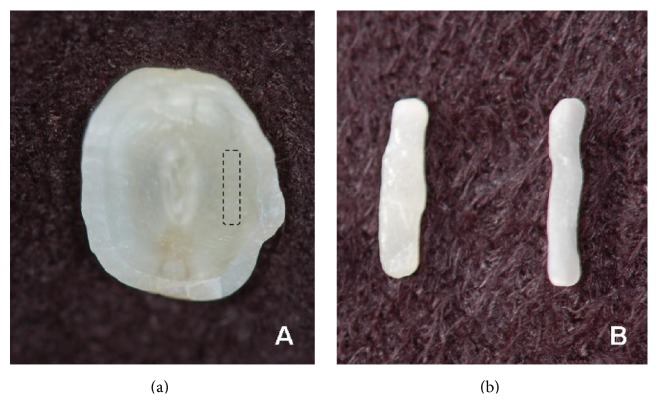
(a) Dental slice cut to obtain the dentinal pins, with outline showing the area from which they were obtained. (b) Dentinal biopins.

An extracted tooth of the same size, shape, and color as tooth 21 (Figure 3) was used to cut the dental fragment and in the subsequent homogenous bonding. The tooth to be used in the homogenous restoration was sterilized in moist heat at 121°C for 15 minutes [16]. The cutting of the dental fragment and the “biopins” was performed under extreme refrigeration with diamond burs (Figure 4).  Figure 5(a) displays the production process of the “biopins,” which was performed by cutting a coronal slice, following the transverse direction of the tooth, in order that the dentinal tubules were perpendicular along the axis of the pin. These were then separated until a cylindrical shape of approximately 1 mm diameter and 4 mm length was obtained (Figure 5(b)).

**Figure 6:**
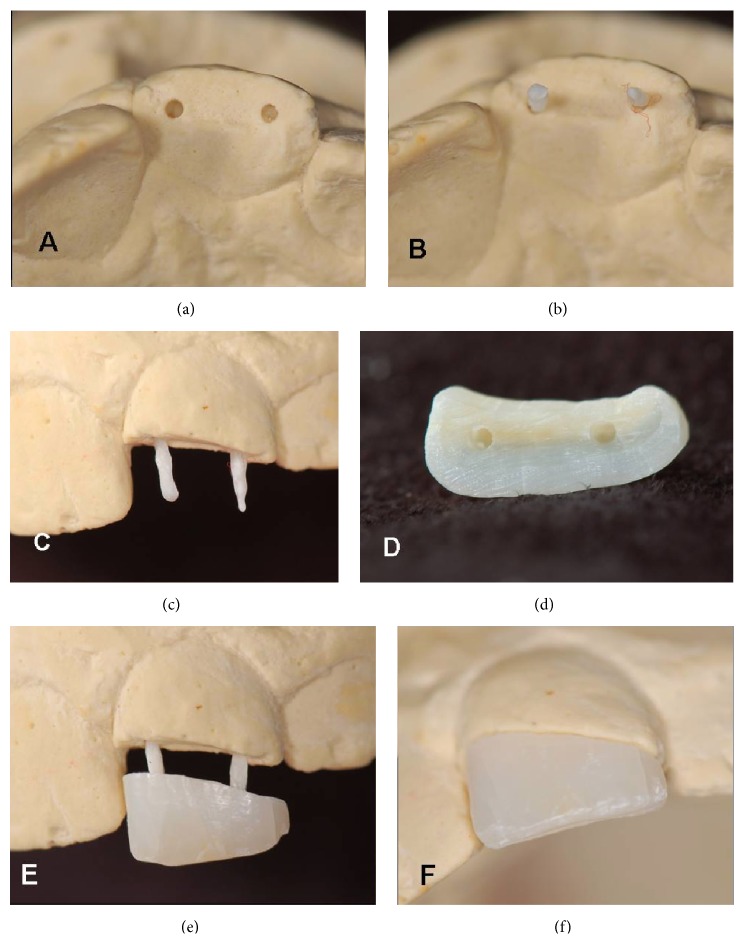
(a) Simulated holes in the model for placement of the biopins. (b) Biopins in place, incisal view. (c) Biopins in place, vestibular view. (d) Holes made in the fragment for adaptation of the biopins. (e) Laboratory sample of the fragment to the biopins. (f) Fragment suitably adapted to the plaster model.

After the cutting and adaptation of the restorative fragment to the model (Figure 4), a drilling simulation was performed to place the biopins in the plaster die (Figure 6(a)) and in the fragment (Figure 6(d)), using a spherical bur (1 mm diameter and a depth of 2 mm) with a low rotation. This was to simulate what would be performed at a later stage with the remainder of the tooth.

**Figure 7:**
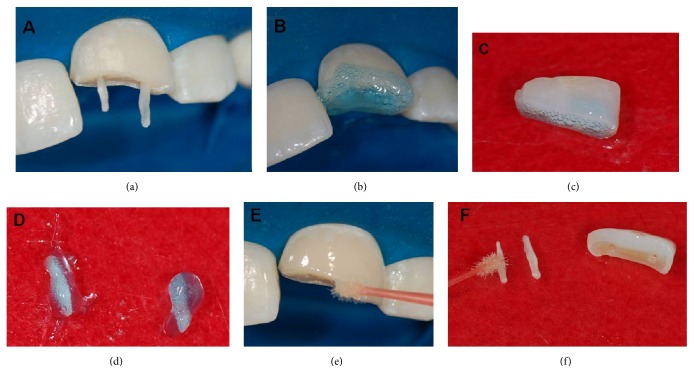
(a) Sample of the biopins in the holes made in the remaining tooth. (b) Acid conditioning of remaining tooth. (c) Acid conditioning of dental fragment. (d) Acid conditioning of the biopins. (e) Application of the primer/adhesive on the remaining teeth. (f) Application of the primer/adhesive in the biopins and dental fragment.

**Figure 8:**
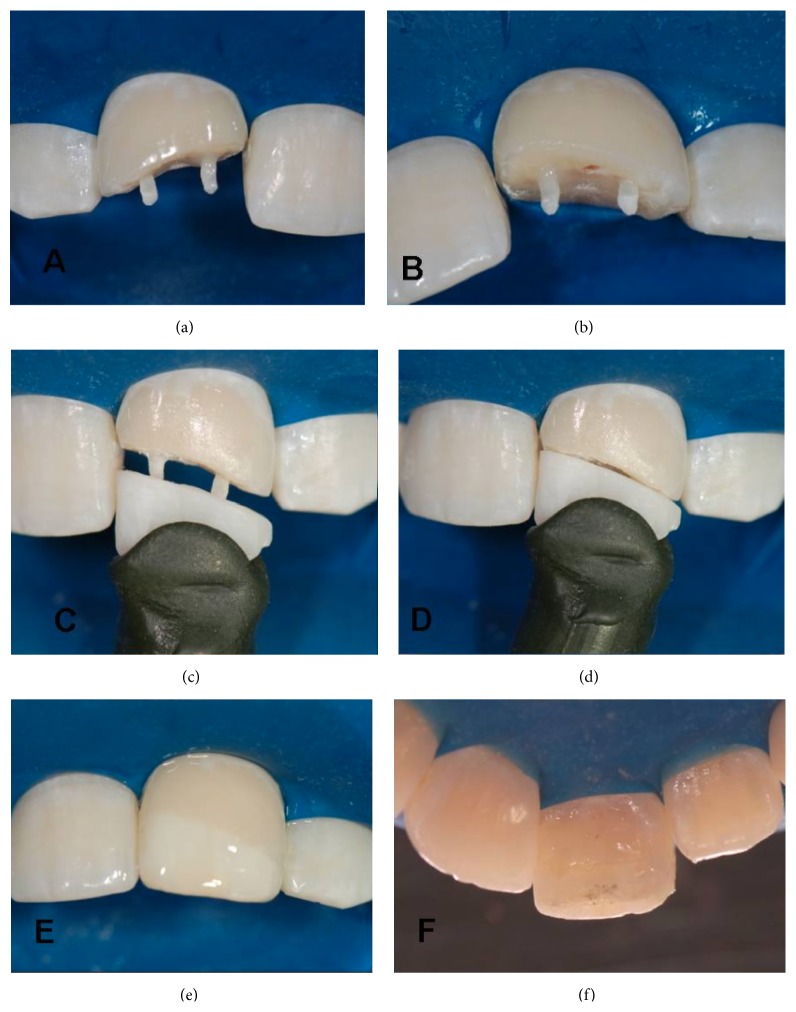
Cemented biopins in the remaining tooth. (a) Vestibular view. (b) Incisal view. (c) Clinical sample of the dental fragment. (d) Fragment adapted to the remaining tooth. (e) Vestibular aspect after bonding the vestibular fragment. (f) Palatal aspect after bonding the fragment.

In the second clinical session, perforation of the remainder of the tooth was conducted, through absolute isolation, for the cementation of the “biopins” with a spherical bur of 1 mm diameter (Figure 7(a)). Subsequently, conditioning treatment with phosphoric acid (37%) was performed for 30 seconds in enamel and for 15 seconds in dentin from the fragment, from the remainder of the tooth, and from the “biopins” (Figure 7). This was followed by application of the adhesive system (Adper Single Bond 2, 3M ESPE, Brazil) and photopolymerization for 20 seconds (Figure 7). After conditioning, the “biopins” were cemented to the dentin with FLOW resin of color A2 (Vigodent, 3M ESPE, Rio de Janeiro, Brazil) and photopolymerized for 40 seconds (Figures 8(a) and 8(b)). Subsequently, the dental fragment was embedded in the dentine pins, cemented with FLOW resin A2 (Figures 8(c) and 8(d)), and photopolymerized for 40 seconds on all sides (Figure 8(f)).

**Figure 9:**
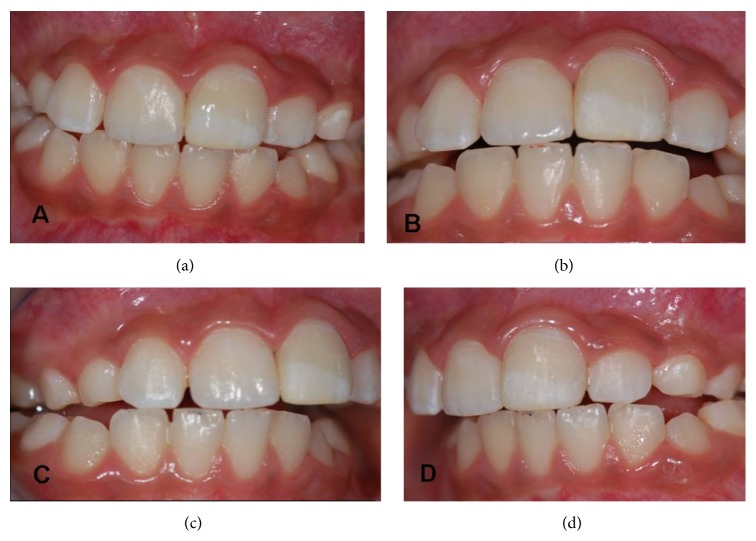
(a) Clinical aspect of the restoration after removal of the absolute isolation. (b) Clinical aspect in protrusion. (c) and (d) Clinical aspects in laterality.

**Figure 10:**
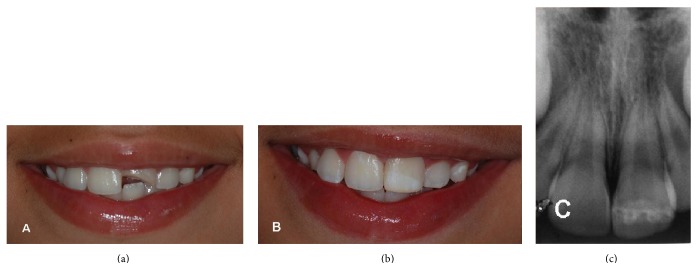
(a) Initial clinical aspect. (b) Clinical aspect shortly after bonding. (c) Final radiographic aspect.

After the cementation of the dental fragment (Figure 9), occlusal adjustment was carried out. Seven days after the conclusion of the homogenous bonding of the fragment, the bevel was constructed at the apparent bond line, with spherical diamond bur number 1014. This was done using light-cured composite resin A1 (Z250, ESPE, USA) in order to produce a satisfactory esthetic result. The newly restored dental surface was then finished and polished (Figure 10).

**Figure 11:**
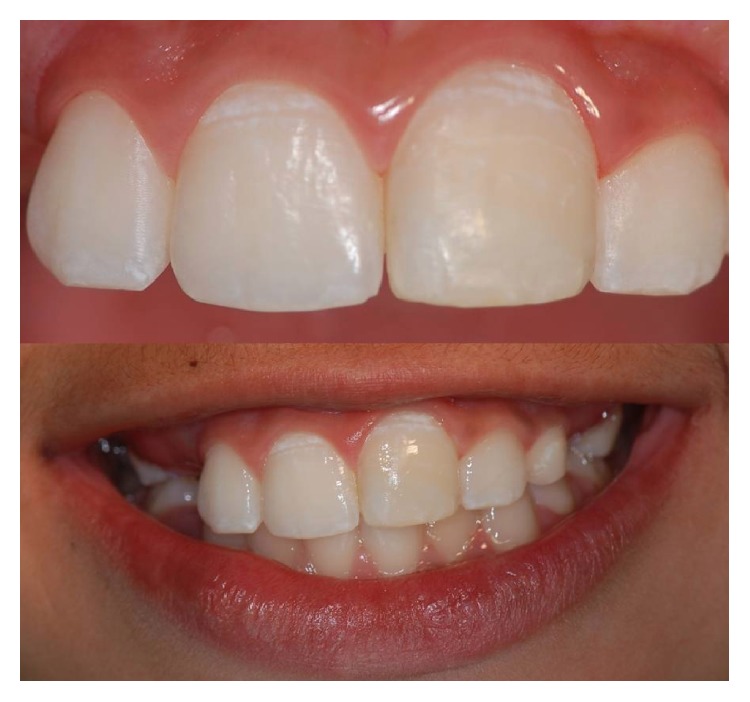
Clinical aspect after 12 months.

The child and their guardian were instructed regarding hygiene, diet, and the need for regular monitoring to preserve the esthetics and functional conditions.  Figure 10(c) displays the aspects of the restoration after one year of clinical follow-up (Figure 11).

## 3. Discussion

The clinical decision regarding the restorative procedure selected for fractured teeth directly affects the treatment prognosis and requires a careful assessment of different factors, such as the extent and pattern of the fracture, the endodontic involvement, and the possibility of using the fragment in the bonding process [25]. In the present case study, homogenous bonding was selected since it was impossible to perform autogenous bonding as the patient no longer had the dental fragment. The association of biopins with homogenous bonding was conducted in an attempt to improve retention of the fragment to the remainder of the tooth. The shape, location, and number of biopins fundamentally depend on the extent of the cavity preparation, the vestibulolingual volume, and the position of the tooth in the arch [8]. The alternative to biopins would be metallic posts threaded in dentin. However, there are disadvantages in using these metallic posts, such as the greater risk of perforating the pulp chamber, inflammatory responses of the pulp, and cracks in the dentin [26].

In previous studies, the dentin post closely resembled root dentin in all physical properties, such as the modulus of elasticity, viscoelastic behavior [24], compressive strength [25], and thermal expansion [26]. Furthermore, dentin has been found to be tougher than most of the other current restorative materials [27]. A dentin post forms a micromechanical homogenous unit with the root dentin, resulting in uniform stress distribution [28, 29]. The similarity in elasticity of a dentin post to root dentin may allow post flexion to mimic tooth flexion so that the post acts as a shock absorber, transmitting only a fraction of the stresses placed on the tooth to the dentinal walls [30]. It is possible that these properties are also applicable to biopins. However, laboratory studies should be performed to test the fracture resistance of fragments and of composite resin restorations associated with biopins.

In the present case study, it was confirmed that the child had already undergone a number of composite resin restorative procedures. These were unsuccessful due to the fact that the patient did not yet have complete permanent dentition. The guides and occlusion were not mutually protected, which led to pressure during jaw movements that could have compromised the stability of prior restorations. After one year of monitoring, the fragment and tooth had correctly adapted their functionality with excellent results in terms of smoothness, esthetic, and maintenance of the incisor guide, as well as physiological and natural characteristics of the dental structure.

In the present case study, the pulp chamber was obliterated with reactionary dentin, which improved the possibility of success of the retention technique using “biopins.” The use of this technique in young patients is significant, since the volume of pulp is an important contraindication [25].

In spite of the previously mentioned advantages of using “biopins,” it is important to refer to a number of their limitations: there is the difficulty of acquisition of extracted teeth; they are difficult to create due to the reduced size; and there is a possibility of perforating the pulp chamber during completion of the technical preparation of the pin hole.

With regard to the ethical aspect, it is necessary to clarify to the patient and/or the parents/guardians that the post is made from duly donated and properly sterilized extracted teeth, thus preventing biosecurity risks. However, a tooth fragment obtained from another patient may be rejected, which is a disadvantage of this technique. The teeth used in biological restoration procedures can be obtained from human teeth banks or from nonprofit institutions, which store and provide teeth for didactic, clinical, and scientific use [31]. The low number of human teeth banks and the limited dissemination of the technique make this an uncommon routine in dental practice [15].

The technique of biological restoration associated with the use of pins created from human dentin (biopins) is another alternative among the different treatment options for vital teeth. This technique is inexpensive and exhibits excellent biocompatibility, as well as maintaining the characteristics of tooth structure such as smoothness, surface brightness, texture, hardness, size, shape, color, resistance, and, consequently, functionality and esthetic.

However, this is a little known technique, and there are very few studies reporting its use in the literature. Therefore, further studies are necessary which demonstrate the mechanical properties of biological restorations and biopins, as well as assessing and monitoring long-term clinical cases.
